# Fracture of the Fabella: An Uncommon Injury in Knee

**DOI:** 10.1155/2015/396710

**Published:** 2015-09-13

**Authors:** Taoufik Cherrad, Jamal Louaste, Hicham Bousbaä, Larbi Amhajji, Rachid Khaled

**Affiliations:** Department of Orthopedic Surgery and Traumatology, Military Hospital Moulay Ismail (HMMI), 50000 Meknes, Morocco

## Abstract

The fabella is a sesamoid bone that may contribute to the stabilization of the posterolateral knee corner and it can very occasionally act as a source of atypical and rare knee pain and functional impairment. Fracture of the fabella is a rare but important clinical entity which may be overlooked clinically and radiographically. However, it causes an intermittent mechanical pain of the knee and it can mistakenly harm another knee pathology like intra-articular loose body. We report a case of a 21-year-old man who was sustaining a fracture of fabella following vehicle accident.

## 1. Introduction

The fabella (Latin for little bean) is a sesamoid bone usually embedded in the lateral head of the gastrocnemius muscle and is present in approximately 10–30% of the population [1].

The fabella may be involved in a variety of pathological entities: fabella syndrome, chondromalacia fabellae, peroneal nerve impingement, fabella dislocation, and fabella fracture which was described for the first time by Sagel on 1932 [2]. To date, dozens of cases of fabella fracture have been described in the literature [3, 4]. Fabellar fractures are rare and may be underdiagnosed. Considering the low prevalence rates, it is quite a diagnostic challenge to properly evaluate these lesions to ensure their adequate management to prevent morbidity.

We report a case of a 21-year-old man who was sustaining a fracture of fabella following vehicle accident.

## 2. Case Report

**Figure 1:**
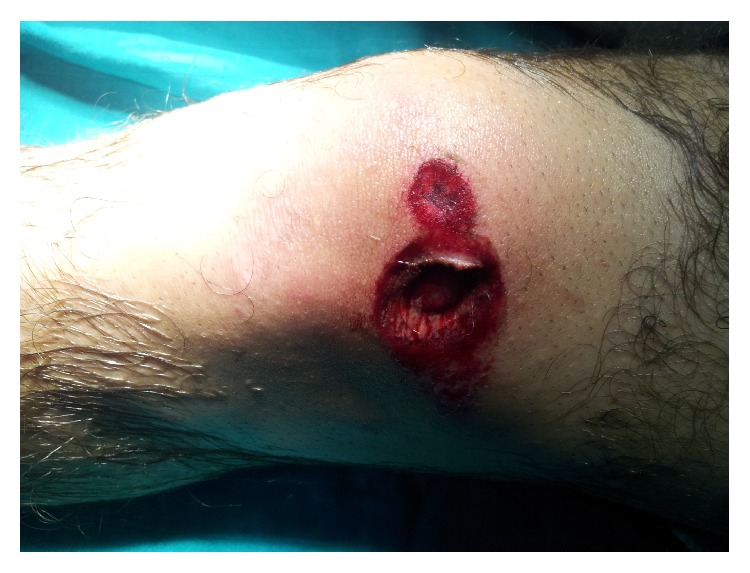
Parapatellar internal wound secondary to crushing.

A 21-year-old male patient presented to the emergency department as a pedestrian who had been struck by an automobile; crushing of the extended knee by the wheel's car caused a parapatellar internal nonarticular wound measuring 2 centimeters (Figure 1). After the initial assessment at the emergency department, he complained of diffuse left knee pain aggravated by mobility and palpation. The distal pulses and the sensation were intact.

**Figure 2:**
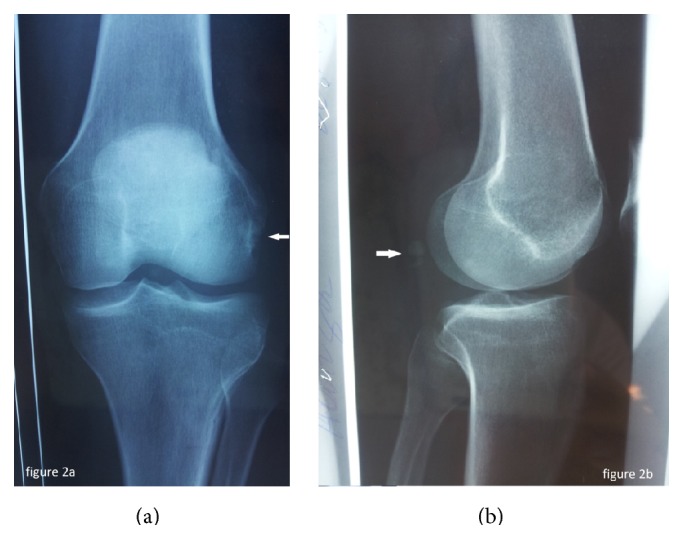
Anteroposterior (a) and lateral (b) plain films of the left knee showing a fabellar fracture.

**Figure 3:**
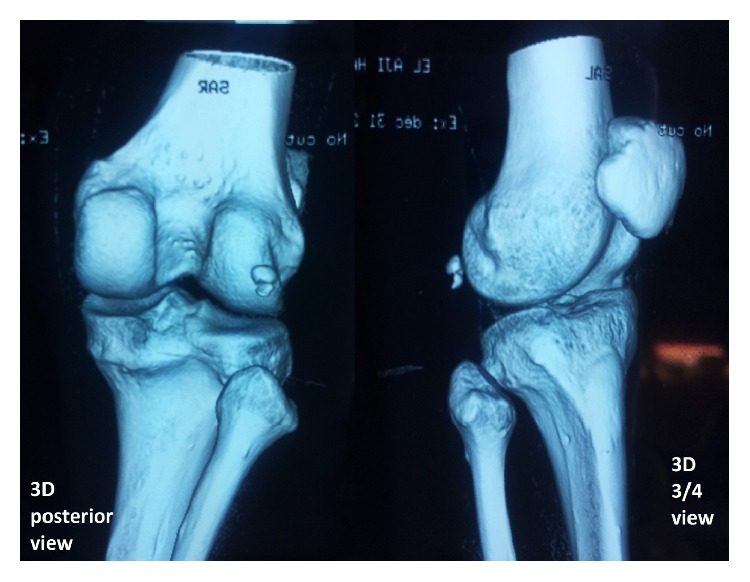
3D-CT reconstruction revealed a fracture of the fabella.

Anteroposterior and lateral plain films of the left knee were then taken for initial evaluation (Figure 2) and revealed a transverse fracture of the fabella. The patient underwent a computed tomography (CT) of the left knee which showed an acute displaced fracture of the fabella (Figure 3).

At the emergency operating room, the patient underwent surgical debridement and under anesthesia the physical examination did not reveal frontal or sagittal plane laxity of the left knee. The fracture of the fabella was managed conservatively. Symptomatic treatment for pain and antibiotic therapy were adopted in the acute setting for this case.

At three-month follow-up, the patient has recovered well and has no current knee complaints.

## 3. Discussion

The fabella is a fibrocartilaginous or ossified sesamoid bone embedded in the tendinous portion of the lateral head of the gastrocnemius muscle, often directly articulating with the posterior surface of the lateral femoral condyle [5].

The development of the fabella remains a source of hypothesis; biomechanical components (including local mechanical stresses associated with locomotion and muscular contraction) and intrinsic genetic factors have been commonly associated with it [6, 7]. The fabella prevalence in the population is estimated at 10 to 30% [6]. The average bony fabellae may measure up to 15 mm [6].

The fabella plays an important biomechanical role in the knee by stabilizing the posterolateral knee corner [6]. The link of the popliteal tendon on the joint capsule and lateral meniscus constitute its principal function [8].

The fabella may be involved in a variety of pathological entities. The fractures of this sesamoid are rare and could be underdiagnosed. It may occur at all ages and appear after direct trauma, like in our case, or after chronic stress forces, such as impingement after total knee replacement [3].

X-ray and especially the lateral plain film can establish the diagnosis. However, when the lesion is suspected; the CT or MRI confirms the fracture and guides an adequate management of care to prevent morbidity mainly related to knee pain and functional impairment [3, 4].

Treatment is usually conservative in the acute phase. Once the fabellar fracture is confirmed, the associated lesions should be sought especially at the lateral compartment of the knee.

The evolution can be characterized by the onset of posterolateral mechanical knee pain; the pain is aggravated by the complete extension and rebound tenderness on the posterior side of the knee; in this situation injecting local anaesthetic and steroid should be performed as first time; alternatively the fabellectomy may resolve the problem [7, 9].

Fracture of the fabella is rare; it happened following direct knee trauma or after chronic stress forces. When this fracture is suspected, the CT or MRI evaluation of the knee may confirm fabellar fracture.

If this entity is under diagnosis, it can simulate several other knee conditions with different clinical presentations, and in the long term it may cause chronic painful knee.
